# Health information orientation and health literacy as determinants of health promotion behaviors in adolescents: a cross-sectional study

**DOI:** 10.3389/fpubh.2024.1522838

**Published:** 2025-01-20

**Authors:** Mi-Ae You, Jeong-Ah Ahn

**Affiliations:** Research Institute of Nursing Science, College of Nursing, Ajou University, Suwon-si, Republic of Korea

**Keywords:** adolescent, health promotion, health literacy, information seeking behavior, socioeconomic disparities in health

## Abstract

**Introduction:**

This cross-sectional study aimed to identify the impact of health information orientation and health literacy on adolescents’ health-promoting behaviors.

**Methods:**

We enrolled 149 middle school students from an urban city in South Korea through convenience sampling. The data was collected in October 2022 using a self-reported questionnaire. Health information orientation was measured using the Health Information Orientation Instrument (Cronbach’s *α* = 0.86). Health literacy was assessed with the Korean Adolescent Health Literacy Scale (KR-20 = 0.66), and health promotion behaviors were evaluated using the Adolescent Health Promotion Scale-Short Form (Cronbach’s *α* = 0.89). Analysis methods included independent *t*-tests, one-way ANOVA, and multiple regression.

**Results:**

Results indicated significant differences in health promotion behaviors based on perceived health status, economic status, primary health-related information provider, and health literacy. Health information orientation showed a strong positive correlation with health promotion behaviors. The factors that influenced health promotion behaviors were health information orientation, primary information provider, economic status, and health literacy.

**Discussion:**

Findings suggest that school and community health promotion programs should engage adolescents and parents to enhance health literacy and proactive information-seeking behaviors for improving the health outcomes of adolescents.

## 1 Introduction

Adolescence is a critical developmental stage marked by significant physical, emotional, and cognitive changes. During this period, adolescents develop enhanced information-processing abilities and are capable of abstract thinking, allowing them to make inferences and process complex information (1). With growing access to various technological devices, including mobile devices and mass media, adolescents frequently seek health information online (2). Establishing healthy behaviors during adolescence through structured health education is crucial for laying the groundwork for a healthy adulthood (3).

Promoting adolescents’ engagement with their health is essential for encouraging both health knowledge acquisition and positive health practices (4). Health promotion behaviors, which are predictive of current health status and future disease risk, develop through complex interactions involving cognitive, social, physical, and emotional factors, as well as individual experiences and personality traits (5). Adolescents’ health promotion behaviors are closely linked to their ability to acquire and understand health-related information, highlighting the need to understand these diverse characteristics to encourage behavioral changes (6).

Health information orientation is defined as an individual’s proactive approach to seeking health information, motivated by a desire to engage with health behaviors (7). Adolescents with a high health information orientation are more likely to gather health-related information from reliable sources, demonstrating a greater readiness to use this information in making health-related decisions (8). This orientation has been shown to positively influence health promotion behaviors, suggesting that adolescents who actively seek health information are also more likely to adopt healthy behaviors (9).

Adolescents access health-related information from various online sources, including search engines, social media platforms, health websites, and mobile apps (10). While credible sources, such as government or medical websites, promote positive health behaviors, unreliable platforms, like certain social media channels, can lead to misconceptions and risky behaviors for adolescents (11). The type and reliability of these sources significantly influence health promotion behaviors, with verified sources fostering healthier practices (10). Guiding adolescents toward credible information is essential to enhancing their health literacy and promoting better health outcomes.

Health literacy, recognized as a crucial asset for managing individual and public health issues, encompasses the ability to find, understand, and apply health information to make informed decisions that improve one’s health (12). For adolescents, health literacy not only relates to positive health behaviors but also mitigates behaviors that threaten their health (13). Research indicates that adolescents with higher health literacy report better health outcomes and are more likely to engage in self-management and preventive health behaviors (14). In contrast, those with limited health literacy face higher risks of negative health behaviors, such as smoking, drinking, and obesity (15). Adolescents’ reliance on online information further amplifies the role of health literacy in their health promotion behaviors, making online health information access a significant factor in their decision-making processes (16).

The cultural context of South Korea may significantly influence adolescents’ health behaviors. High academic pressures often limit time for health-promoting activities such as exercise or adequate sleep (17). Additionally, the heavy reliance on technology and social media shapes adolescents’ health information-seeking behaviors, while strong parental involvement remains a key factor in guiding health-related decisions (18).

While some research has examined the link between health literacy and health promotion behaviors in adolescents (19), studies on the combined impact of health information orientation and health literacy on these behaviors remain limited. Additionally, most existing studies have been conducted in Western contexts, leaving a gap in understanding how these factors influence health behaviors among adolescents in South Korea. This study addresses these gaps by investigating the interplay between health information orientation, health literacy, and contextual factors in shaping adolescents’ health promotion behaviors.

## 2 Methods

### 2.1 Study design

This study employed a descriptive, cross-sectional design.

### 2.2 Study population

Study participants were middle school students with no communication impairments who understood the study objectives and voluntarily consented to participate in the study. The participants belonged to a single region in South Korea and were selected using convenience sampling. We enrolled 149 middle school students. Using the G*Power version 3.1.9.2 software (Heinrich Heine University, Düsseldorf, Germany) for a *post hoc* analysis to determine the sample size adequacy and power of the study, the sample size of this study reached a power (1-*β*) of 84.0%, with the medium effect size of 0.15 of multiple regression analysis in this study, and a 2-sided significance level of 0.05.

### 2.3 Study variables

#### 2.3.1 General characteristics

Data were obtained regarding the participants’ general characteristics, including age, gender, economic status, academic achievement, subjective health status, and primary health-related information providers. Economic status was categorized as “upper” or “mid/lower” as perceived by the adolescents. Academic achievement was classified as “high,” “medium,” or “low,” while subjective health status was categorized as “poor,” “average,” or “healthy.” For the primary health-related information providers, participants selected one of three options: “parents,” “healthcare practitioners,” or “friends.”

#### 2.3.2 Health information orientation

To measure health information orientation, we used the Health Information Orientation Instrument developed by Basu and Dutta (7). The instrument consists of nine items, each rated on a 5-point Likert scale ranging from “strongly disagree” (1 point) to “strongly agree” (5 points). Higher scores indicate greater health information-seeking. Cronbach’s *α* was 0.88 at the time of development (7) and 0.86 in this study.

#### 2.3.3 Health literacy

Health literacy was measured using the Korean Adolescent Health Literacy Scale (KHLS-Teen) developed by Jang (20). This instrument consists of 16 items, each rated as “correct” (1 point) or “incorrect” (0 point). Higher scores indicate better health literacy, with ≤10 points considered inadequate health literacy and ≥ 11 points considered adequate health literacy. The KR-20 was 0.75 at the time of development (20) and 0.66 in this study.

#### 2.3.4 Health promotion behaviors

Health promotion behaviors were assessed using the Adolescent Health Promotion Scale- Short Form (AHP-SF) developed by Chen et al. (21). The instrument consists of 21 items, with each item scored on a 5-point Likert scale ranging from “strongly disagree” (1 point) to “strongly agree” (5 points). Higher scores indicate a higher degree of practice in health promotion behaviors. Cronbach’s *α* was 0.91 at the time of development (21) and 0.89 in this study.

### 2.4 Data collection

Data was collected between October 1 and 31, 2022. We administered the questionnaire to middle school students currently attending a school in an urban city in South Korea. We obtained the cooperation of the principal and school nurses after explaining the aims and procedure of the study. Once we determined the possible dates and times for the survey, we distributed the questionnaires and had the students complete the questionnaire, place it in an anonymized envelope, and leave it in the collection box. Participants in the questionnaire survey were given a small voucher as a token of appreciation for their participation in the study.

### 2.5 Data analysis

This study employed IBM SPSS version 28.0 (IBM Corp., Armonk, NY, USA). Participants’ general characteristics, health information orientation, health literacy, and health promotion behaviors were analyzed using descriptive statistics comprising frequency, percentage, mean, and standard deviation. We used independent *t*-tests and one-way analysis of variance to analyze the differences in health promotion behaviors according to the participants’ general characteristics, and the post-hoc analysis was conducted using the Scheffé test. We calculated Pearson’s correlation coefficients to explore the relationships between health information orientation, health literacy, and health promotion behaviors. Finally, we performed multiple regression analyses to identify the factors affecting health-promotion behaviors in adolescents.

### 2.6 Ethical considerations

Institutional Review Board (IRB) approval was obtained from Ajou University (IRB No. AJOUIRB-SB-2022-382). Before study inclusion, the purpose and procedure were explained to the participants. Considering that the participants were adolescents, we provided them with sufficient time to make decisions regarding their participation in the study. Additionally, for any unclear parts of the explanation, we offered a thorough Q&A opportunity and provided further clarification as needed. A home letter was sent to students who were willing to participate, and the survey was administered to students whose parents had understood the study and gave their permission to participate voluntarily.

## 3 Results

### 3.1 Participants’ general characteristics

Among the 149 participants, 88 (59.1%) were girls and 61 (40.9%) were boys. The mean age was 14.81 (± 0.39) years. Seventy-six participants (51.0%) reported their subjective health status as average, 58 (38.9%) as healthy, and 15 (10.1%) as poor. The primary providers of health-related information were parents for 83 participants (55.7%) and healthcare practitioners for 56 participants (37.6%) (Table 1).

**Table 1:** Participants’ general characteristics (*N* = 149).

Characteristics	*n* (%)
Gender	
Boys	61 (40.9)
Girls	88 (59.1)
Grade	
8	27 (18.1)
9	122 (81.9)
Academic achievement	
High	40 (26.8)
Medium	80 (53.7)
Low	29 (19.5)
Subjective health status	
Poor	15 (10.1)
Average	76 (51.0)
Healthy	58 (38.9)
Economic status	
Upper	38 (25.5)
Mid/lower	111 (74.5)
Primary health-related information provider	
Parents	83 (55.7)
Healthcare practitioners	56 (37.6)
Friends	10 (6.7)

### 3.2 Participants’ health information orientation, health literacy, and health promotion behaviors

Participants’ mean scores for health information orientation and health literacy were 31.47 ± 6.27 out of 45 and 12.54 ± 2.57 out of 16, respectively. Also, the mean score for health promotion behaviors was 66.80 ± 13.52 out of 105, indicating a moderate level of health promoting behaviors among adolescents (Table 2).

**Table 2:** Participants’ health information orientation, health literacy, and health promotion behaviors (*N* = 149).

Variables	Mean ± SD	Minimum	Maximum	Range
Health information orientation	31.47 ± 6.27	17	45	9–45
Health literacy	12.54 ± 2.57	4	16	0–16
Health promotion behaviors	66.80 ± 13.52	31	101	21–105

### 3.3 Differences in health promotion behaviors according to general characteristics

Among the general characteristics, there were statistically significant differences in health promotion behaviors according to the participants’ subjective health status, economic status, and the primary provider of health-related information. Health promotion behavior score was significantly higher in the healthy group than in the average group for subjective health status (*F* = 4.79, *p* = 0.010), and in the “upper” economic status group than in the “mid/lower” economic status group (*t* = 3.31, *p* = 0.001). In addition, there was a significant difference in the score of health promotion behavior according to the primary health-related information provider (*F* = 3.34, *p* = 0.038). Health promotion behavior was significantly higher in the adequate health literacy group than in the inadequate health literacy group (*t* = 2.48, *p* = 0.014) (Table 3).

**Table 3:** Difference in health promotion behaviors according to general characteristics (*N* = 149).

Characteristics	Mean ± SD	t/F	*p* (Scheffé)
Gender			
Boys	67.26 ± 14.29	0.34	0.732
Girls	66.49 ± 13.03		
Grade			
8	67.41 ± 13.74	0.26	0.799
9	66.67 ± 13.52		
Academic achievement			
High	68.73 ± 14.24	2.60	0.078
Medium	67.66 ± 12.48		
Low	61.81 ± 14.51		
Subjective health status			
Poor ^a^	63.97 ± 14.68	4.79	0.010
Average ^b^	64.17 ± 13.22		(b < c)
Healthy ^c^	70.99 ± 12.74		
Economic status			
High	72.87 ± 13.70	3.31	0.001
Mid/lower	64.73 ± 12.87		
Primary health-related information provider			
Parents	68.85 ± 13.49	3.34	0.038
Healthcare practitioners	65.28 ± 13.92		
Friends	57.40 ± 13.94		
Health literacy			
Inadequate (*n* = 32)	61.65 ± 15.86	2.48	0.014
Adequate (*n* = 117)	68.21 ± 12.52		

The a,b,c are for Scheffe test.

### 3.4 Relationships between health information orientation, health literacy, and health promotion behaviors

As shown in Table 4, health promotion behaviors showed a statistically significant positive correlation with health information orientation (*r* = 0.57, *p* < 0.001).

**Table 4:** Relationships between health information orientation, health literacy, and health promotion behaviors (*N* = 149).

	Health information orientation	Health literacy	Health promotion behaviors
	*r* (*p*)	*r* (*p*)	*r* (*p*)
Health information orientation	1		
Health literacy	0.45 (0.076)	1	
Health promotion behaviors	0.57 (< 0.001)	0.15 (0.072)	1

### 3.5 Factors affecting health promotion behaviors

To determine the factors affecting health-promoting behaviors, we performed multiple regression analyses using health orientation, health literacy, and general characteristics that showed significant differences (subjective health status, economic status, and primary health-related information provider) as independent variables (Table 5). We confirmed that the variance inflation factor was not ≥10 (range, 1.03–4.21), and the tolerance was ≥0.1 (range, 0.24–0.98), suggesting that there were no problems with multicollinearity. When we tested the independence of the residuals, the Durbin-Watson statistic was 2.05, which is close to 2, suggesting no problems with autocorrelation.

**Table 5:** Factors affecting health promotion behaviors (*N* = 149).

Variables	B	SE	*ß*	*t*	*p*
Subjective health status (moderate)^1^	1.21	2.95	0.05	0.41	0.681
Subjective health status (healthy)^1^	5.50	3.01	0.20	1.83	0.070
Economic status (upper)^2^	5.98	1.99	0.19	3.01	0.003
Health-related information provider (parents)^3^	8.21	3.48	0.30	2.36	0.020
Health-related information provider (healthcare practitioners)^3^	6.34	3.59	0.23	1.77	0.080
Health information orientation	1.10	0.14	0.51	8.04	<0.001
Health literacy (adequate)^4^	4.81	2.12	0.15	2.27	0.025
*R*^2^ = 0.44, *F* = 15.96, *p* < 0.001					

Reference group ^1^poor, ^2^mid/lower, ^3^friends, ^4^inadequate.

In the multiple regression analysis, the factors affecting participants’ health promotion behaviors were economic status, primary health-related information provider, health literacy, and health information orientation. Health promotion behaviors were significantly higher in adolescents with higher health information orientation (*β* = 0.51), with parents rather than friends as the primary health-related information provider (*β* = 0.30), with upper rather than mid-lower economic status (*β* = 0.19), and with adequate health literacy (*β* = 0.15). The explanatory power of the model was 44.0% (*R*^2^ = 0.44). These results indicate that improving health information orientation and literacy, alongside parental involvement and addressing socioeconomic disparities, could significantly enhance adolescents’ health promotion behaviors.

## 4 Discussion

This study investigated the impact of health information orientation, health literacy, and sociodemographic factors on adolescents’ health promotion behaviors. The results highlight critical elements that influence health promotion behaviors among adolescents, offering valuable insights for enhancing health-related interventions.

Health information orientation was the most significant factor affecting health promotion behaviors in our study. Adolescents with a higher health information orientation scored significantly better in health promotion behaviors. Health information orientation refers to the extent to which an individual is willing to search for health information (7). It suggests that a proactive attitude to obtaining health information may facilitate healthier choices (22). This finding aligns with previous studies indicating that individuals who actively seek health information are better equipped to make informed decisions that support long-term health outcomes (23). With the recent development of mobile services and technologies, a number of healthcare consumers actively seek health information via mobile devices (24). Especially in South Korea, the high reliance on technology and social media may intensify adolescents’ engagement with online health information sources compared to adolescents in countries with less advanced digital infrastructure. Adolescents with a strong health information orientation can be motivated to seek social communication channels that provide information about health-related topics and look for methods to use the information for health behaviors (25). Encouraging adolescents to actively seek credible health information could play an important role in strengthening their health promotion behaviors.

Similar to international studies, our findings confirm that health literacy emerged as a significant predictor of health promotion behaviors. Adolescents with adequate health literacy demonstrated higher engagement in health-promoting activities compared to those with inadequate literacy levels. This result reinforces the notion that understanding health information is crucial for implementing healthy behaviors. Adolescents with adequate health literacy are better positioned to comprehend the benefits of health-promoting actions and apply health information effectively in their daily lives (26). Adolescents with high health literacy levels are more likely to engage in health promoting behaviors and communicate effectively with health professionals (16). As suggested by existing literature, improving health literacy through targeted educational programs could be instrumental in equipping adolescents with the skills necessary to adopt healthier lifestyles (27). Adolescents with adequate health literacy are more adept at navigating healthcare systems, identifying credible health information sources, and utilizing available resources effectively. This ability can directly translate into improved health outcomes and more consistent health-promoting behaviors. In addition, among middle school students in an urban region, 21.5% showed inadequate health literacy in our study. This result differs from a survey of middle school students in rural villages (13) in which 67.5% had inadequate health literacy. This indicates regional differences in health literacy, with rural students showing lower health literacy than urban students. Therefore, it is crucial to evaluate health literacy in adolescents by region and provide tailored education to improve students’ health literacy for better health promotion behaviors. As a good example, the World Health Organization’s Health Promoting schools framework has been successful and is widely accepted as a whole-school approach to providing students with a supportive environment to develop health literacy (28).

Economic status was another influential factor in determining health promotion behaviors among adolescents. Those from higher economic backgrounds exhibited greater involvement in health-promoting behaviors than those from lower economic backgrounds. This result may be reflective of the additional resources and opportunities available to adolescents in higher economic groups, such as access to healthier foods, recreational sports facilities, and healthcare resources. This finding is consistent with the social determinants of health framework, which posits that economic status substantially affects access to health-promoting resources (15). In addition, the competitive academic environment and associated costs in South Korea may exacerbate disparities, as higher-income families often have more resources to invest in extracurricular activities and health-promoting environments. Policies from schools and communities aimed at reducing health disparities should consider socioeconomic factors and prioritize equitable access to necessary resources for health promotion among adolescents.

The primary source of health-related information also had a significant impact on health promotion behaviors. Adolescents who identified their parents as their main health information providers engaged in healthier behaviors than those who relied on friends. This finding highlights the role of family in shaping adolescents’ health attitudes and behaviors. Parents, who often provide more reliable and accurate information, appear to positively influence their children’s health choices (29). Studies from Western countries have also highlighted the role of healthcare professionals as key health information providers for adolescents (30), whereas our study emphasizes the strong influence of parents in the Korean context. This reflects the nature of Korean culture, where family plays a central role in shaping adolescents’ behaviors. Educational initiatives that involve parents and caregivers could strengthen adolescents’ health promotion behaviors by reinforcing reliable health messages and supporting positive health behaviors within the family (31).

This study represents the multifaceted nature of health behaviors and emphasizes the need for comprehensive approaches in health promotion efforts. Programs targeting adolescents should adopt an integrated strategy that promotes health literacy, encourages a proactive approach to health information, and considers socioeconomic barriers. By addressing these factors together, healthcare providers and educators can foster sustainable health-promoting behaviors in adolescent populations.

### 4.1 Limitations

Despite the study’s strengths, several limitations should be considered. First, the cross-sectional design limits the ability to establish causal relationships. Future studies could address this limitation by employing longitudinal designs to better understand how health information orientation and literacy impact health behaviors over time. Second, we recruited a convenience sample of middle school students from a single urban area, which may not represent adolescents in rural or suburban settings. Urban adolescents may have greater access to health-related information and resources, potentially influencing the findings. Therefore, caution should be required when generalizing the results to adolescents in other regions or those with different socioeconomic and cultural contexts. Future research should include more diverse samples from various geographic and socioeconomic backgrounds to enhance generalizability. Third, the reliance on self-reported measures may introduce bias, as adolescents’ subjective assessments of their socioeconomic status, health, and behaviors may not align with objective data. Future research could benefit from a mixed-methods approach that combines quantitative measures with qualitative insights to capture a more comprehensive understanding of adolescents’ health behaviors. Fourth, the relatively low reliability of the health literacy scale (Cronbach’s *α* = 0.66) may have influenced the findings. This suggests potential variability in the responses and highlights the need for further refinement of the scale for future use.

## 5 Conclusion

This study highlights the importance of health information orientation, health literacy, and socioeconomic factors in shaping adolescents’ health promotion behaviors. These findings emphasize the need for targeted and tailored interventions that foster health literacy, support proactive health information engagement, and address socioeconomic disparities to promote healthier lifestyles among adolescents. Through collaborative efforts involving families, schools, and healthcare providers in communities, it can enhance the health and well-being of young populations more effectively.

### 5.1 Implications

This study highlights the importance of addressing health information orientation and health literacy in clinical and community health settings to support adolescents’ health-promoting behaviors. School nurses, clinical nurses, and healthcare educators can play a key role in developing programs that enhance adolescents’ engagement with health information, especially by involving parents as primary health information providers to reinforce accurate health messaging. For public policy, training programs for parents could focus on equipping them with skills to effectively communicate credible health information and model healthy behaviors to their adolescents. Educators should integrate health literacy and critical health information-seeking skills into school curriculums, teaching adolescents to evaluate the credibility of online health sources. Given the significant association between socioeconomic status and health-promoting behaviors, targeted interventions that consider economic disparities may improve equitable access to health resources. Integrating health literacy education into routine adolescent care and school-based health programs can empower adolescents with the knowledge and skills necessary for healthier decision-making, potentially reducing future healthcare utilization associated with poor health behaviors. These strategies support a proactive approach in clinical and educational settings to foster sustainable health behaviors among adolescents, ultimately contributing to their long-term health and well-being.

## Data Availability

The raw data supporting the conclusions of this article will be made available by the authors, upon reasonable request.
